# Deep Ocean Temperature from the Stratus Ocean Reference Station (85 °W, 20 °S), 2012–2025

**DOI:** 10.1038/s41597-026-07680-8

**Published:** 2026-06-30

**Authors:** Yu Gao, Albert Plueddemann, Robert A. Weller, Sebastien Bigorre

**Affiliations:** Woods Hole Oceanographic Institution, 266 Woods Hole Road, Woods Hole, MA 02543-1050 USA

## Abstract

Long-term deep ocean temperature monitoring is crucial for understanding the ocean’s role in climate variability and storage of heat in the deep ocean. Observation of the ocean surface is relatively accessible via both *in-situ* and remote sensing; however, continuous, high-temporal resolution, decade-long temperature records from abyssal depths face the technical challenges of sustained deep ocean deployments. The addition of calibrated, internally-recording temperature sensors to deep-ocean moorings not far from the sea floor provides a means of making high temporal resolution temperature observations. Quality controlling and merging records from successive mooring deployments results in a decade-long time series. Our work has been to optimize approaches for ensuring data quality and continuity in multi-year deep ocean temperature datasets. Here we show a comprehensive processing framework that yields 13 years (2012 to 2025) of continuous temperature measurements at approximately 4200 to 4500 meters depth from the Stratus Ocean Reference Station near 22 °S, 85 °W, 1500 km off the coast of Chile in the Southeast Pacific. Our framework incorporates timing checks, automated spike detection, systematic multi-sensor validation, statistical validation, human-in-the-loop quality control, and merging protocols. This framework establishes reproducible standards for processing long term oceanographic observations from multiple deployments. For the Stratus data set, the result is a unique, decade-long abyssal temperature record with quantified uncertainties that constitutes a benchmark time series for evaluating the realism of deep ocean temperature in models.

## Background & Summary

Long-term continuous observation of deep ocean temperature can help understand the ocean’s role in climate and ocean heat content variability. Compared to the surface of the ocean, deep ocean observations are rare, especially decade-long continuous temperature at abyssal depth (typically defined from 3,000 to 6,500 meters). About a decade ago, the physical oceanography community recognized the need for additional temperature observations from the deep oceans to drive improvement of model representation of the downward mixing of heat and other properties into the deep ocean. Such measurements could be assimilated into ocean models and used to assess the realism of climate model outputs. Bottom mounted instrument packages and existing moorings provide opportunities to add observations of deep ocean temperature. Tracey *et al*.^1^ examined a four-year record of deep temperature observations in the Drake Passage and Meinen *et al*.^2^ reported a decade of near-bottom temperature observations in the Argentine Basin, both from pressure‐equipped inverted echo sounder (PIES) moorings. Uchida *et al*.^3^ show a 13 year record of near-bottom temperature from a CTD mounted 34 m above the seafloor.

To further increase deep temperature observations, OceanSITES^4^, the international collaborative effort to collect sustained time series observations at open ocean sites under the Global Ocean Observing System (GOOS), launched an effort to coordinate deployment of temperature recorders on existing deep ocean moorings. The Upper Ocean Processes Group at Woods Hole Oceanographic Institution (WHOI) joined this effort and committed to deploy pairs of temperature recorders on Ocean Reference Stations^5^. A station in the North Pacific Ocean (47 °N, 160 °E) produced a decade-long continuous temperature at abyssal depth from 2010 to 2024^3^. This paper focuses on the work at another one of these Ocean Reference Stations to obtain, quality control, document, and share deep ocean temperature time series.

The Stratus Ocean Reference Station (ORS) is located near 22 °S, 85 °W, 1,500 km west of northern Chile. Deployment of deep-ocean instruments at the Stratus site started in 2012 using two Conductivity, Temperature and Depth Instruments (CTDs) on the mooring. Sampling began on 2012-05-28 02:30:00 and data collected up to 2025-03-18 11:00:00 UTC are considered. On a roughly annual basis, a new surface mooring was deployed and the existing mooring recovered. Due to occasional mooring and instrument problems, some short gaps exist between 2012 and 2025. Overall, gaps represent only 0.07% of the data set. The longest gap is 105.5 hours (4.4 days) between 2014-03-03T12:30 and 2014-03-07T22:30, which occurs between the recovery of Stratus 12 and the deployment of Stratus 13. Temperature observations were collected using Seabird SBE-16 and SBE-37 instrument pairs mounted at a fixed depth above the sea floor. Each sensor was calibrated prior to deployment. Although the instruments record temperature, conductivity and (in some cases) pressure, only the temperature data have been processed and released to date. Temperature sensing was found to be stable, while conductivity and salinity computed from it were not stable in these deep deployments, and a means to correct them is not yet in hand.

Instrument depths range from 4191 to 4526 meters. Instrument depth is determined by subtracting the instrument height above the bottom from the water depth. Instrument height is a fixed depth of 39 m, determined from summation of the mooring components from the anchor to the load bar containing the instruments. The water depth is obtained from a shipboard fathometer and is corrected for sound speed variation. This value is unique to each deployment and is denoted as “water_depth_from_ship_corrected_m” in the metadata.

Temperature data are processed and quality-controlled using six major steps: timing checks, automated spike detection, systematic multi-sensor validation, statistical validation, expert validation, and merging protocols. The processed dataset includes temperature, latitude, longitude, instrument depth and timestamps. The dataset spans multiple mooring deployments. The moorings are deployed for year-long intervals with a standardized design, traceable sensor calibrations and overlapping “burn-in” (validation) periods to ensure data continuity and quality^5^. Data gaps are present between some deployments, and minor gaps may also arise during periods of instrument failure and other operational issues. The deployments were merged into a continuous time series as described in the metadata. Data gaps are filled with standard fill values and are therefore readily identifiable.

The merged Stratus time series represents the longest *in-situ* deep ocean temperature record in the region, with potential uses including studies of ocean heat content and validation of ocean models. The data contributes to broader observational programs such as OceanSITES and GOOS.

## Methods

### Instrumentation

Temperature data were collected using pairs of either SBE-16 or SBE-37 microCAT sensors^6^. For each deployment, a pair of identical sensors were deployed 39 meters above the mooring anchor on the sea floor. All sensors were factory-calibrated prior to deployment preparation. In preparation for deployment, all sensors underwent a laboratory burn-in period during which the instruments were run continuously to verify sensor stability, clock synchronization, and data logging functionality.

Recovered data from each instrument was processed separately prior to merging. Instrumentation was first deployed in 2012 on the 12th setting of the Stratus mooring (Stratus 12) and data was processed through the 22nd setting (Stratus 22). Table 1 summarizes the sensor models, instrument depths, sampling intervals, and measured parameters for each deployment. All parameters were included in initial processing steps, but only temperature data were fully quality controlled for inclusion in the merged time series.

**Table 1:** Instrumentation used in each deployment, including sensor model, deployment depth, sampling interval, and measured variables.

Deployment	Year	Model	Depth (m)	Sampling frequency	Variables
Stratus 12	2012	SBE16	4500	30 min	Temperature, Salinity
Stratus 13	2014	SBE16	4502	30 min	Temperature, Salinity
Stratus 14	2015	SBE37	4471	5 min	Temperature, Salinity, Pressure
Stratus 15	2016	SBE37	4526	5 min	Temperature, Salinity, Pressure
Stratus 16	2017	SBE37	4495	5 min	Temperature, Salinity, Pressure
Stratus 17	2018	SBE37	4526	5 min	Temperature, Salinity
Stratus 18	2019	SBE37	4229	5 min	Temperature, Salinity, Pressure
Stratus 19	2021	SBE37	4191	5 min	Temperature, Salinity, Pressure
Stratus 20	2022	SBE37	4222	5 min	Temperature, Salinity, Pressure
Stratus 21	2023-02	SBE37	4199	5 min	Temperature, Salinity, Pressure
Stratus 22	2023-12	SBE37	4237	5 min	Temperature, Salinity, Pressure

All sensors were manufactured by Sea-Bird Scientific.

### Data processing

#### Time check and truncation

We begin by checking the potential timing errors. To verify instrument clock accuracy, a timing mark was created both before deployment and after recovery. This involved briefly submerging the instruments in cold water to induce a sharp temperature change or “spike” and independently logging the start and end times of the event for comparison with the instrument’s internal timestamps. Potential timing errors are addressed by comparing the spike times in the data to the deployment and recovery instrument log. If the offset is greater than one sampling interval (30 minutes for Stratus 12 and 13, 5 minutes for Stratus 14 to 22), it indicates misalignment in time, and the data is linearly interpolated to a new time base to synchronize instruments. For the Stratus 12–22 deployments, we did not find time misalignment, so no interpolation was done.

Data set truncation is necessary to remove data prior to deployment and after recovery. After the anchor is dropped from the ship, it takes several hours to fall to the sea floor. Tracking the acoustic release just above the anchor has shown that the anchor is down and at rest within 4 hours after anchor drop. Specifically, we take the following steps for data truncation:

- Read anchor-over time and release-fired time from metadata.
- Truncate the dataset to start 4 hours after anchor-over time and end at release-fired time (note, the 4-hour delay allows time for the anchor to drop to the bottom and the mooring line to settle into a stable configuration).
- Plot and inspect temperature time series for both instruments.
- Ensure that truncation has successfully excluded data from before deployment and after recovery.

**Fig. 1:**
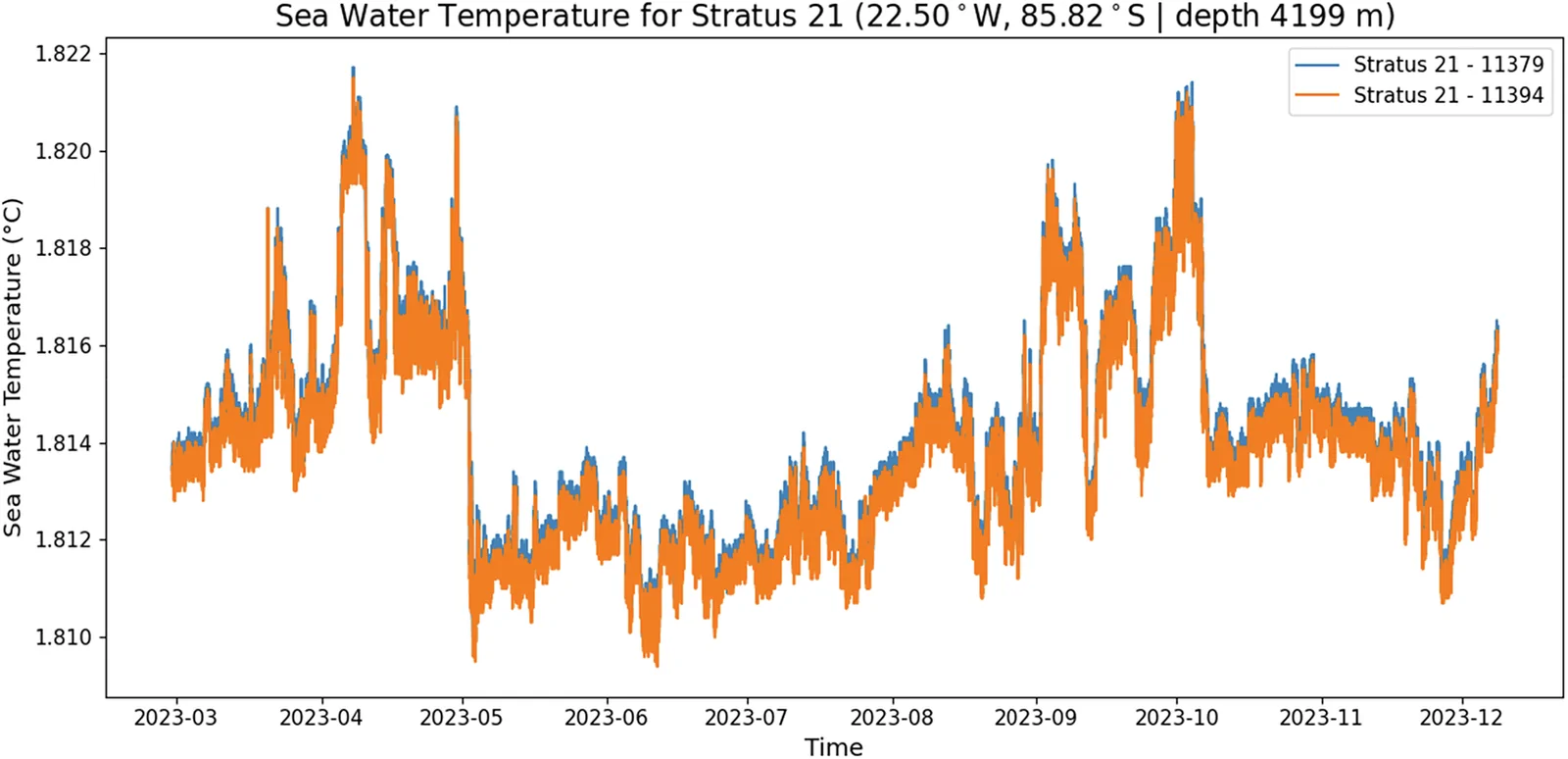
Temperature comparison of co-located instruments during Stratus 21 deployment (2021–2022) showing SBE37 sensors 11379 and 11394 at 4199 m depth. The survey location is at 22.50 °W, 85.82 °S. The instrument depth is at 4199 meters.

An example data set from Stratus 21, after time check and truncation, is shown in Fig. 1. Data from both instruments are plotted to aid in data evaluation. For Stratus 21, the correlation values (0.997 for temperature) demonstrate excellent inter-instrument agreement.

#### Spike removal

Spike removal is a standard component of oceanographic quality control where we remove the anomalous outliers in the data, since spurious values can arise from electronic noise or sensor instability and lead to misleading variability in the dataset^7^. We identify and remove spikes from time series data using a rolling mean and standard deviation within a 2-hour window around each data point. Data points that deviate from the rolling mean by more than 3 times the standard deviation are considered spikes and replaced with fill values.

#### Uncertainty estimates

To estimate temperature measurement uncertainty, we compared temperature data from co-located sensors deployed at the same depth. The difference statistics are computed after spike removal to avoid contamination by known bad points. When data from both instruments is available, we compute the mean and standard deviation of temperature for each sensor, and for the difference between sensors. Cross-sensor error is defined as one standard deviation of the difference between two records from duplicate sensors^8^. The cross-sensor differences are an indication of sensor precision and can also be used to inform quality control processing and relative sensor drift that may occur during deployment.

The difference statistics are stored as metadata in the NetCDF files with the following attributes for the temperature variable:

- std_single_sensor: Standard deviation of the time series from a single sensor
- std_sensor_diff: Standard deviation of the difference between two co-located sensors
- mean_sensor_diff: Mean of the difference between two co-located sensors.

There are several sources of uncertainties in instrument depth. The synthetic mooring components may elongate during deployment due to sustained mooring tension. The anchor may tip over or sink into the seafloor. Although 84 seventeen-inch glass balls directly above the instruments provide substantial vertical tension, the mooring may tilt. Water depth from the shipboard fathometer may be in error. Each error is of order 1–2 m. If assumed independent, the overall depth uncertainty depth is about 3 m.

#### Human-in-the-Loop (HITL) quality control

A standard “catalog” of time series plots for temperature, salinity, and pressure like Fig. 1 are created for each deployment, including both raw and quality-controlled plots for all measured variables. Human-in-the-loop (HITL) quality control involved domain expert review of these plots to determine whether additional data removal or adjustment was necessary. We assessed each time series for signs of sensor drift, sensor failure, and outliers that were not captured by automated checks. This manual inspection is particularly effective for identifying long-term drift, and the long drift after deployment in pressure and conductivity was detected in this check (not shown). Because pressure drift affects instrument depth measured by its pressure sensors, we used a fixed instrument depth based on shipboard bathymetry and mooring geometry rather than time-varying pressure measurements.

#### Standardized metadata

With the method described above, we developed a standardized pipeline to process the raw data and ensure consistent metadata, data quality and temporal consistency. Our metadata follow the OceanSITES data format^9^ and are compliant with Climate and Forecast (CF) Metadata Conventions, version 1.8 (CF-1.8). The metadata structure includes categories describing (i) deployment and platform information (site code, cruise names, deployment/recovery times, institutional affiliations), (ii) geospatial and temporal coverage (latitude, longitude, depth, start and end times, merge points), (iii) instrumentation (model, manufacturer, serial numbers, mounting, instrument depth), (iv) measured variables (sea water temperature with associated CF standard names, units, and uncertainty metrics), (v) data processing and quality control (processing version, date, conventions, quality comments), and (vi) administrative and citation information (license, acknowledgements, project website, dataset summary).

#### Merging deployments

The creation of continuous time series from sequential mooring deployments required careful handling of temporal overlaps and data gaps. Overlaps result from the standard practice of deploying the new mooring prior to recovery of the old mooring^5^. Gaps may result from sensor failures prior to mooring recovery, or inability to follow the standard practice due to weather or other constraints on field operations.

For each deployment transition, the following protocol was implemented:

- Instrument Selection: When multiple instruments were used for a single deployment, we selected the instrument providing the most complete record and showing the smallest difference from the preceding and following deployment to ensure smooth transitions across deployments.
- Overlap Handling: When temporal overlap existed between consecutive deployments, optimal merge points were identified by the smallest mean differences over rolling 6-hour windows. This approach ensured smooth transitions between time series while preserving the natural variability characteristics of each instrument.
- Gap Management: For non-overlapping deployments, data from each instrument are joined end-to-end and we fill the gap with “NaN” to create a continuous time series. The merge occurred at the start timestamp of each additional dataset, and all merge points were documented in the metadata for transparency and reproducibility.
- Sampling Rate: When instruments operated at different sampling frequencies, higher-frequency data were resampled to lower frequency to maintain temporal consistency across the merged dataset. Stratus 12 and 13 have a sampling interval of 30 min. Stratus 14–22, with a sampling interval of 5 min, were resampled to 30-min intervals. Because the SBE-16 instruments make discrete samples at 30 min intervals, we used resampling, not averaging, of the SBE-37 instruments to create a consistent time series at 30 min intervals.
- Metadata Merging: We retained the complete deployment-specific metadata (e.g., anchor coordinates, instrument serial numbers, deployment and recovery dates, cruise identifiers) derived from a consistent metadata structure across all deployments. We recalculate geospatial and temporal coverage attributes at the merged dataset level to accurately represent the complete record’s minimum and maximum latitude, longitude, depth and time. Merge points were explicitly defined in the metadata to document the transitions between deployments, ensuring traceability of all contributing datasets. This approach preserves both the integrity of deployment-level information and the usability of the merged dataset within the OceanSITES framework.

## Data Record

This deep ocean mooring temperature dataset^10^ is archived at Woods Hole Oceanographic Institution (WHOI) Data Library and Archives at https://doi.org/10.26027/DATAKX5CQK. The repository contains two files: (1) **ReadMe.txt**, a plain-text file (2 KB) providing basic metadata, and (2) **stratus_temperature_2012_2025.nc**, a NetCDF file (1.0 MB) containing deployment metadata and temperature time-series data collected from the Stratus mooring between 2012 and 2025. The NetCDF file includes variables for temperature, depth, and time, with all timestamps recorded in UTC.

## Technical Validation

The six major processing steps in creating the merged temperature dataset are described above. In this section the outcome of selected assessments relating to data validation and interpretation are presented in more detail.

### Dataset continuity

**Fig. 2:**
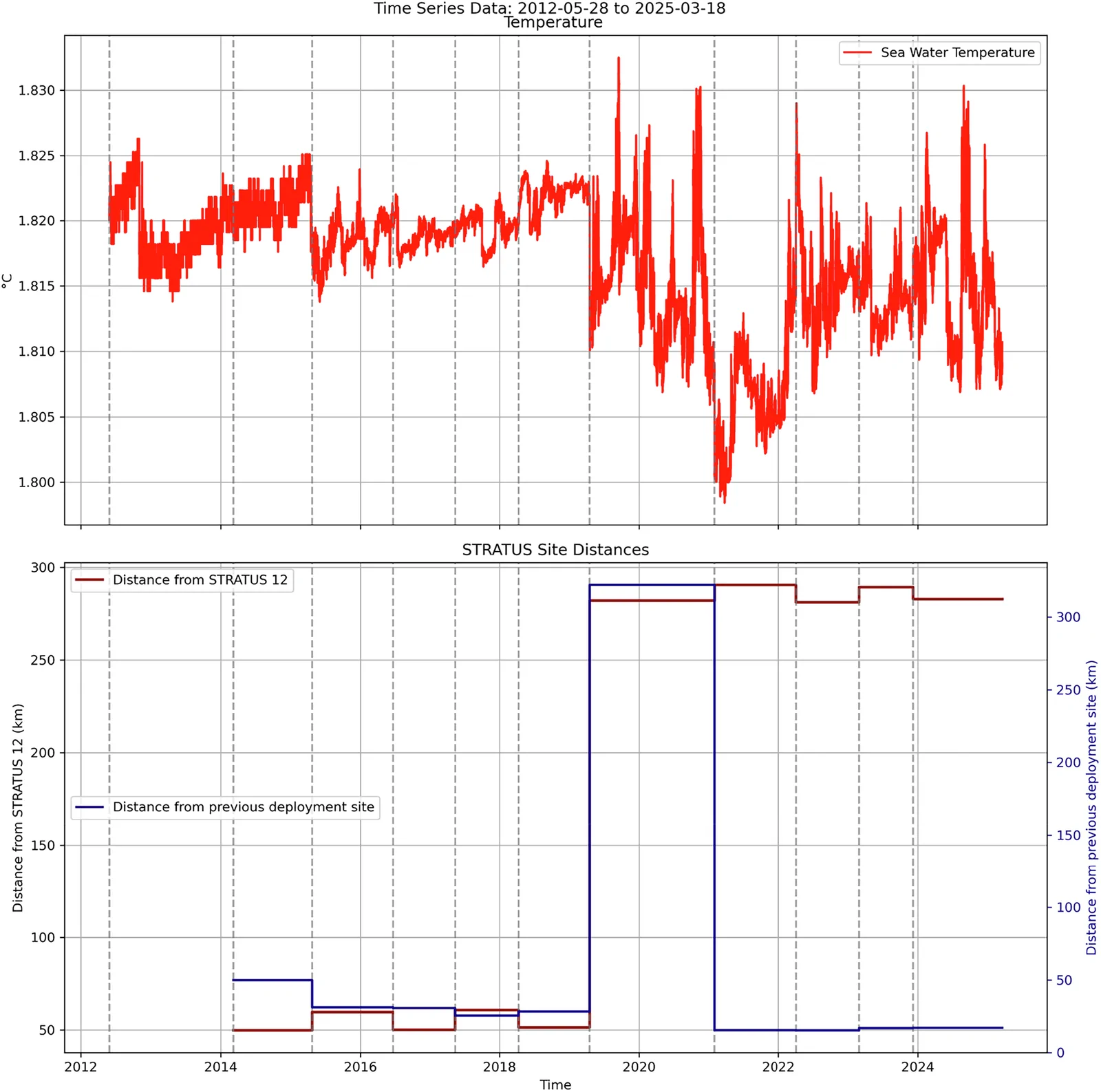
Complete merged temperature dataset from Stratus 12 to Stratus 22 deployments (2012–2025). The upper panels display a continuous time series of deep-sea water temperature at approximately 4200–4500 m depth. The lower panel shows the spatial displacement of each deployment relative to the original Stratus 12 location (red line) and to the immediately preceding deployment (blue line). Despite inter-deployment distances reaching several hundred kilometers in some cases, the majority of successive deployments remained within close proximity to each other.

The complete merged temperature record (Fig. 2) illustrates the full scope of the dataset, with coverage across all deployments from 2012 to 2025. Gaps were confined to brief periods between deployments, and no prolonged gaps were observed. This continuity underscores the unique value of the record for long-term deep-ocean studies.

**Fig. 3:**
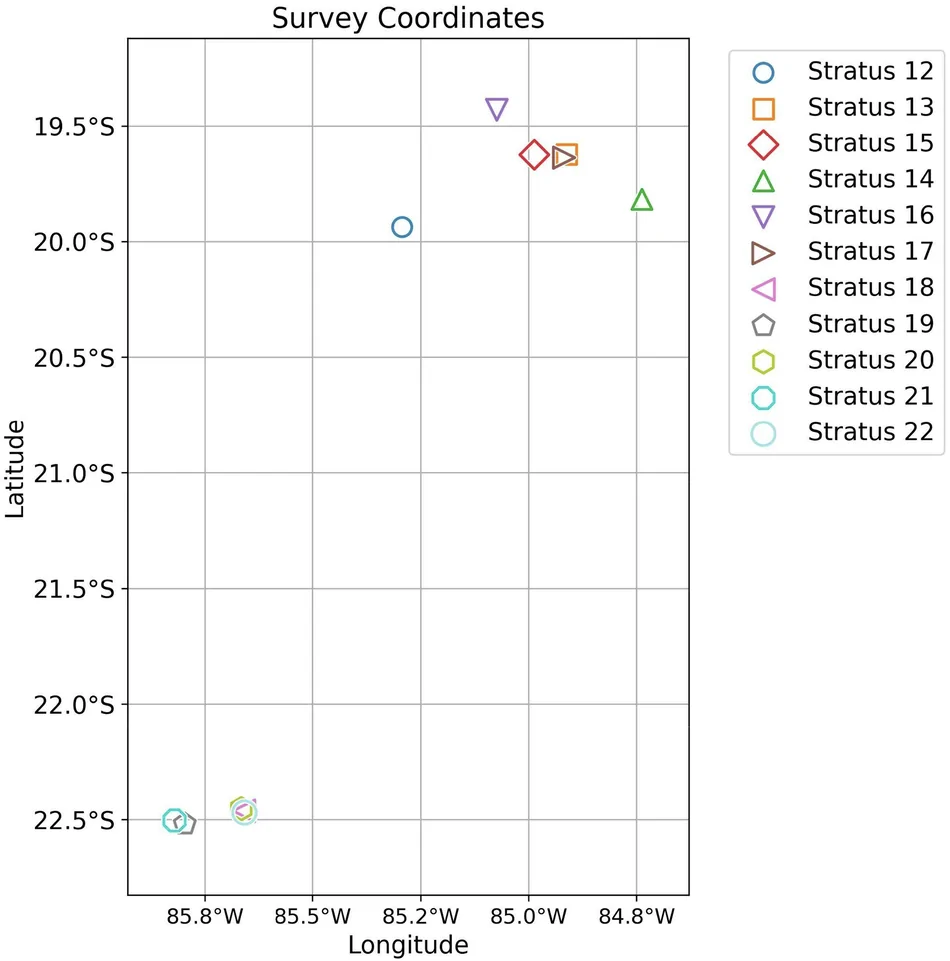
Deployment locations for Stratus 12 through Stratus 22. Each colored marker indicates the mooring position during a given deployment. While some deployments were separated by up to several hundred kilometers, most were redeployed in proximity to each other, ensuring that successive records sampled the same deep-sea environment. The consistency of location minimizes the influence of spatial variability on the merged dataset.

Instrument locations for each deployment are shown in Fig. 3. Deployment of a surface mooring is a day-long process, with the ship slowly steaming towards the anchor site target. That target site is identified during a bottom survey as a large enough, relatively flat area with a depth consistent with the mooring design. At the Stratus site, the bottom topography was challenging, and subsequent mooring sites were intended to be within ~50 km of the Stratus 12 site. The goal is to deploy the new mooring close to the existing mooring to enable intercomparison and continue sampling close to the initial target for the Stratus ORS. However, strongly ridged bottom topography and variable wind, wave, and surface current conditions together with challenges to ship handling at low speed and work on deck, would lead to the decision to deploy over an area of suitable flat bottom rather than towing closer to the target site. After initial deployments, the presence of heavy fishing activity and entanglement of the mooring with fishing gear drove the decision in 2018 to a locus of anchor sites further to the south-southwest. As this work at the Stratus ORS is part of a pilot effort to obtain more observations in the context of very few extant time series of deep ocean temperature, little is known about either the frequency or wavenumber spectra of deep ocean temperature. Because the mooring array was redeployed up to 300 km from the original Stratus 12 site in subsequent years, the successive records reflect the broader deep-sea environment of the region and users should consider the spatial variability when interpreting the merged time series. Position (latitude, longitude) information is included with the merged time series.

### Time check

**Fig. 4:**
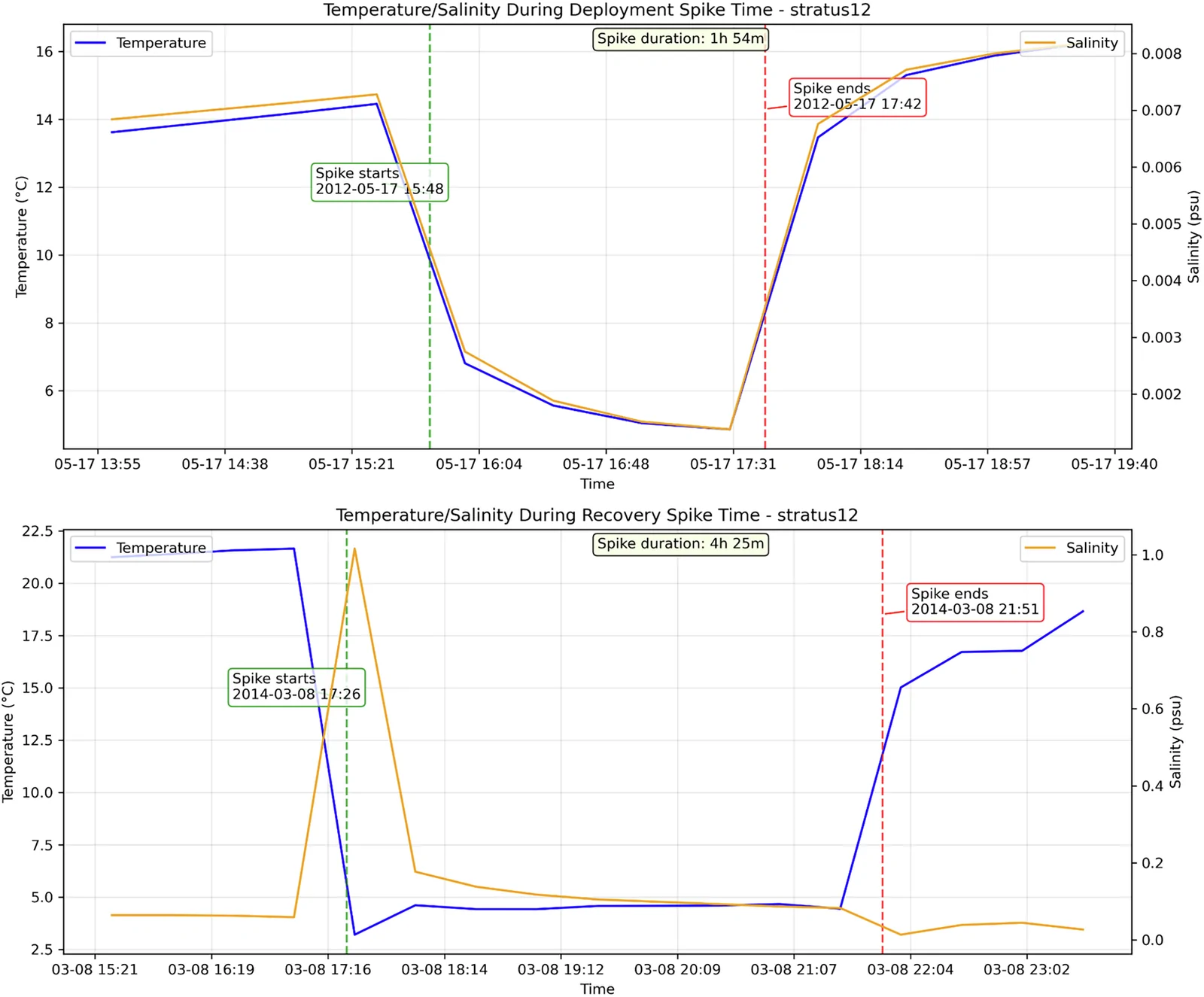
Examples of temperature and salinity anomalies detected during deployment (top) and recovery (bottom) of Stratus 12 SBE16-1876. These short-duration spikes, lasting only a few hours, are associated with handling and stabilization of the instruments rather than environmental variability. Such periods were flagged automatically and excluded from the final dataset, ensuring that only stable, *in-situ* records are retained.

Routine clock checks during deployment and recovery (Fig. 4) revealed no measurable timing drift, confirming that instrument clocks were maintained throughout all deployments. An example of how we validate the time check is below. The recorded time of the temperature spike during deployment was 2012-05-17 15:48, while the time logged by the field team was 2012-05-17 15:48, indicating no timing offset. Similarly, during recovery, the recorded spike time was 2014-03-08 17:26, matching the logged time of 2014-03-08 17:26. This consistency confirms that the instrument clocks remained accurate throughout the deployment period.

### Spike removal

Across all the deployments, the automated spike (outlier) detection removed less than 1% of the temperature observations (Table 2). These points correspond to isolated sensor malfunctions and were excluded from the final dataset. Their removal had negligible impact on overall variability or long-term trends. Note that the fraction of points removed is not a selection criterion.

**Table 2:** Spike removal statistics for Stratus deployments.

Deployment	Instrument Serial Number	Fraction of points removed (%)
Stratus 12	SBE16 1876 (1879)	0.6918 (0.3103)
Stratus 13	SBE16 1875 (1873)	0.2777 (0.0202)
Stratus 14	SBE37 10600 (10601)	0.0065 (0.0065)
Stratus 15	SBE37 12257 (11394)	0.0064 (0.0032)
Stratus 16	SBE37 10600 (10601)	0.0042 (0.0021)
Stratus 17	SBE37 11394 (12257)	0.0084 (0.0065)
Stratus 18	SBE37 10600 (10601)	0.0058 (0.0037)
Stratus 19	SBE37 11379 (11394)	0.0089 (0.0073)
Stratus 20	SBE37 10600 (10601)	0.0073 (0.0042)
Stratus 21	SBE37 11394 (11379)	0.0037 (0.0061)
Stratus 22	SBE37 10600 (10601)	0.0059 (0.0052)

For each deployment, statistics for the primary instrument used in the merged dataset are reported first, followed by those for the secondary (non-selected) instrument in parentheses.

### Error estimation

On each mooring, we deploy two instruments providing overlapping measurements that allow direct consistency checks. The paired records show a high degree of agreement, confirming that relative sensor drift and calibration offsets remained negligible. One example comparison from Stratus 21 is shown in Fig. 1, with a correlation coefficient of 0.997 for temperature.

Cross-sensor error analysis provides quantitative assessment of measurement precision and relative bias across all deployments (Table 3). Estimates below 0.002 °C indicate that they are indistinguishable within the instrument’s measurement uncertainty. The standard deviation of individual sensors ranges from 0.001 °C to 0.005 °C, while cross-sensor differences show standard deviations between 0.001 °C and 0.007 °C. Mean sensor differences are typically within ± 0.001 °C, indicating indistinguishable systematic bias between paired instruments. These statistics confirm that the temperature measurements have high precision, with uncertainties well within acceptable limits for deep ocean observations.

**Table 3:** Temperature measurement error statistics for selected deployments.

Deployment	Deployment Year	Single Sensor	Sensor Std Dev Difference	Mean Difference
		Std Dev (°C)	(°C)	(°C)
Stratus 12	2012	0.002	0.003	−0.001
Stratus 13	2014	0.001	0.002	−0.003
Stratus 14	2015	0.002	0.002	0.0001
Stratus 15	2016	0.001	0.001	0.001
Stratus 16	2017	0.001	0.002	0.000
Stratus 17	2018	0.001	0.002	−0.002
Stratus 18	2019	0.005	0.007	0.001
Stratus 19	2021	0.004	0.006	0.000
Stratus 20	2022	0.003	0.005	0.000
Stratus 21	2023-2	0.002	0.003	0.000
Stratus 22	2023-12	0.005	0.007	0.000

Values computed from paired sensors deployed at the same depth after spike removal and quality control. The single sensor standard deviation difference is calculated by subtracting the standard deviation of the secondary sensor from that of the primary sensor. The mean difference is calculated by subtracting the mean temperature of the secondary sensor from that of the primary sensor. Since the instrument accuracy is ±0.002 °C, all error estimates were rounded to three decimal places. Values that are round to zero are reported as 0.000, following standard scientific practice.

To assess absolute bias (calibration drift), we evaluated 13 post calibration records for 4 SBE-37s between 2013 and 2023. Accounting for the time between calibrations, they indicated a mean drift rate of ~0.01 m°C/month. This is consistent with the assessment of drifts in deep Argo temperature sensors^11^ and well within the manufacturer’s specification of 0.2 m°C/month. It is worth noting that the manufacturer’s accuracy specification (e.g. 0.002 °C for the SBE-37) is based on laboratory evaluations. For deep sea deployments, pressure sensitivity could be a cause for temperature bias of 0–0.002 °C per 4000 dbar^12^.

Together, the results of continuity, quality control, and error estimate demonstrate that the dataset is both internally consistent and robust against timing or sensor-related artifacts, providing strong evidence of calibration stability and inter-deployment coherence.

## Usage Notes

This decade-long temperature record is useful for studies of deep ocean variability, heat content, and model validation. However, users should be aware of the following limitations: 1. Spatial Variability: Although deployments were generally co-located, some were separated by 50 to 300 km. Users should consider potential spatial variability when interpreting the merged time series. 2. Data Gaps: Brief gaps exist between deployments, which may affect analyses requiring continuous records. These gaps are clearly marked with fill values. 3. Salinity and Pressure: While salinity and pressure data were collected, they are not included in the public dataset due to pressure sensor drift issues.

## Data Availability

This deep ocean mooring temperature dataset is archived at Woods Hole Oceanographic Institution (WHOI) Data Library and Archives and is publicly available at the following URL: [https://doi.org/10.26027/DATAKX5CQK].

## Code availability

This deep ocean mooring temperature processing code is available publicly on the following GitHub repository: https://github.com/yugaophd/ORS-processing. (Verified accessible on 11/17/2025). The processing code was written in Python (version 3.10). The repository includes scripts for data quality control, averaging, and NetCDF file formatting used in this study.
